# Effect of Surface Integrity on Hot Fatigue Life of Ti_2_AlNb Intermetallic Alloy

**DOI:** 10.3390/ma14174841

**Published:** 2021-08-26

**Authors:** Yanju Wang, Yi Zhou, Aixue Sha, Xingwu Li

**Affiliations:** 1 Materials Evaluation Center for Aeronautical and Aeroengine Applications, AECC Beijing Institute of Aeronautical Materials, Beijing 100095, China; 13693573517@139.com (A.S.); 13911206573@139.com (X.L.); 2 Key Laboratory of Advanced Titanium Alloys, AECC Beijing Institute of Aeronautical Materials, Beijing 100095, China; ChinaMonday@163.com

**Keywords:** surface integrity, turning, hot fatigue life, Ti_2_AlNb, residual stress

## Abstract

The effect of surface integrity on the hot fatigue performance of Ti_2_AlNb alloy was investigated. A turning process was used to prepare the standard specimens for hot fatigue tests. The surface integrity characterization and axial fatigue tests were performed. The results show that the influence of surface roughness on the hot fatigue performance of the Ti_2_AlNb alloy is a secondary factor. The compressive residual stress and enhanced microhardness in the surface layer has a significant effect on the hot fatigue life and they are dominant in the hot fatigue behavior of the Ti_2_AlNb alloy. Through the investigation on the characteristics of the fatigue fractures, the fatigue propagation process was significantly suppressed because of the strong residual compressive stress and microhardness distribution on the surface layer of the Ti_2_AlNb specimen.

## 1. Introduction

With the continuous development of the aerospace industry, the performance requirements of materials are increasingly high, especially for aero-engine materials. The development of high-temperature-resistant structural materials directly restricts the development of engines. In recent years, the third generation Ti_2_AlNb alloy with high Nb content was proposed and studied. The Ti_2_AlNb intermetallic alloy is a new type of Ti-Al intermetallic compound with ordered orthogonal structure O phase as the main phase composition [1,2], which has high specific strength, good room temperature plasticity, good oxidation resistance, high strength at high temperature, fracture toughness and excellent creep resistance [3,4,5]. With the increasing application of Ti-Al alloys in high-temperature aerospace parts, many studies have been reported on the fatigue performance of the Ti-Al alloy [6,7], but few on Ti_2_AlNb, so that the thermal fatigue research of Ti_2_AlNb alloys is urgently needed.

Relevant data studies have shown that the integrity of the machined surface has an important impact on fatigue life and service performance of the parts [8,9]. Surface integrity is a comprehensive evaluating system for the machined surface state and its evaluation indexes include surface roughness, microstructure, microhardness distribution, residual stress distribution, etc. For the research on the influence law of surface roughness on fatigue life, most scholars believe that the increase of surface roughness is unfavorable to the fatigue performance of parts. However, some scholars have obtained some different conclusions in their research [10,11,12,13]. Du et al. studied the relationship between machined surface integrity and fatigue properties of the TC21 titanium alloy. The surface integrity indexes that have a great impact on fatigue performance include the difference between surface roughness, surface texture and the distribution difference of microhardness. The residual stress distribution and metallographic structure of surface materials have little effect on fatigue properties [14]. Wang et al. studied the effect of machined surface integrity on fatigue properties of a nickel-based single crystal alloy. It was found that surface roughness has a significant effect on fatigue performance. The greater the surface roughness, the worse the fatigue performance [15].

However, some other scholars have different conclusions. Maiya studied the influence form of surface roughness on fatigue performance and found that surface roughness only affects the initiation of fatigue crack [16]. There are other factors that also affect fatigue life. In addition to surface roughness, residual stress distribution also has a significant effect on fatigue properties. When the residual stress of machined surface is very small, there is a strong relationship between surface roughness and fatigue life of the parts. In some cases with large surface roughness, fatigue life also increases significantly. It is found that micromechanical characteristics such as residual stress also have a significant impact on fatigue life [17,18,19]. The residual compressive stress gradient distribution layer introduced by machining on the surface of the part material can form a “neutralization” effect with the load stress borne by the part during the service of the part, so as to reduce the average load stress, draw up the generation and propagation of fatigue cracks and improve fatigue life [20,21]. There are other factors that affect fatigue life.

In this article, three groups of standard specimens were prepared with different turning parameters. Then, the surface integrity characteristics, including surface texture, surface roughness, surface stress concentration, residual stress distribution and micro hardness distribution, were tested, evaluated and analyzed. The fatigue fractures of the specimens were observed by SEM (scanning electron microscope). Meanwhile, the fatigue tests results were discussed according to the measurement results of surface integrity characteristics.

## 2. Experimental Section

### 2.1. Material Properties

The material studied in this paper is the hot isostatic pressing Ti_2_AlNb-based alloy with a nominal composition of Ti-22Al-23Nb-2(Mo,Zr) (at.%). Its detail chemical composition is shown in Table 1.

**Figure 1:**
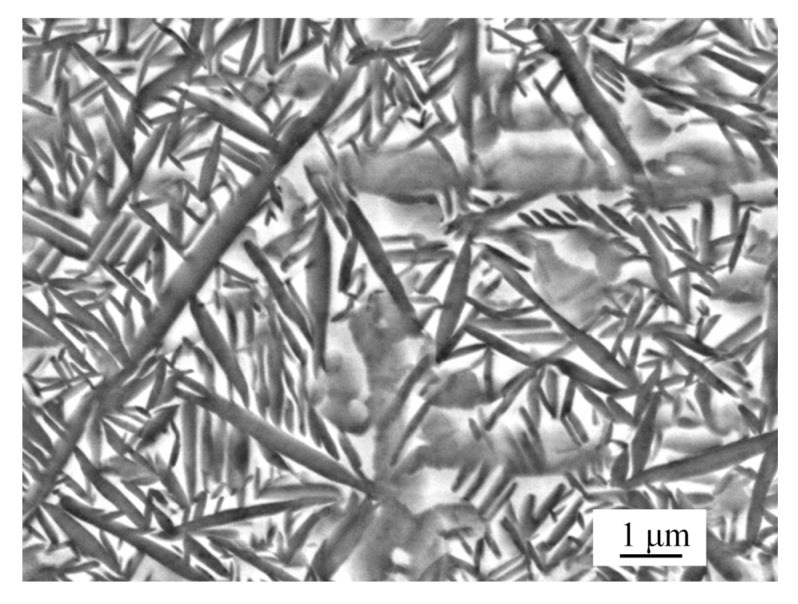
Material microstructure imaged by SEM (10 K×).

The matrix microstructure of the Ti-22Al-23Nb-2(Mo,Zr) alloy was characterized by the O-Ti_2_AlNb phase with lath or needle structure and the B_2_ phase with grey colony structure [10], which is shown in Figure 1. The yield stress of the Ti-22Al-23Nb-2(Mo,Zr) alloy was determined to be 1003 MPa at room temperature. In order to express it concisely, the Ti-22Al-23Nb-2(Mo,Zr) alloy is referred to as the Ti_2_AlNb alloy hereafter in this paper.

### 2.2. Specimen Preparation

**Figure 2:**
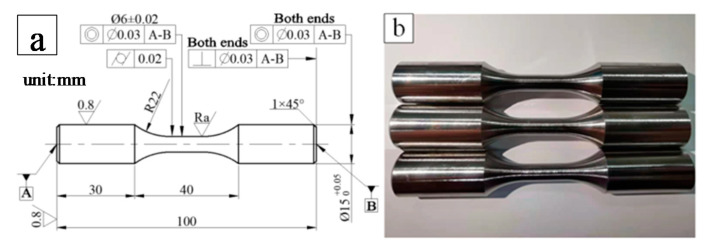
Configuration of fatigue specimen. (**a**) geometry design plan; (**b**) photo of specimens.

**Figure 3:**
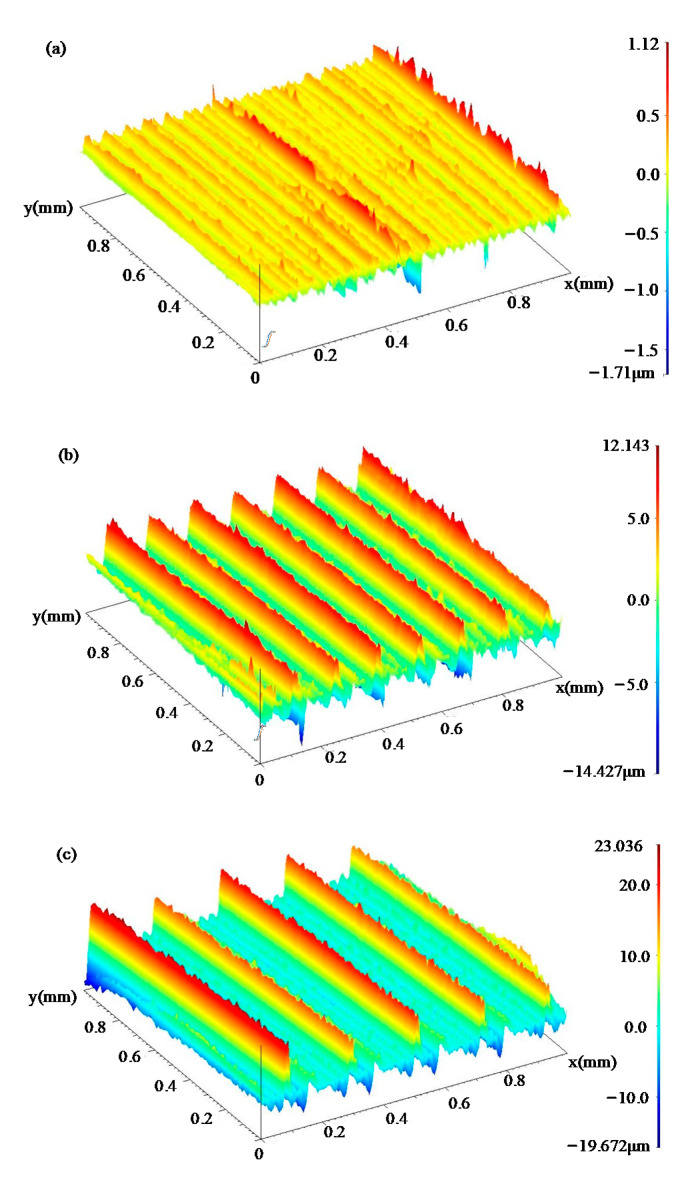
Surface topography of the specimens with different processing parameter: (**a**) Group 1, (**b**) Group 2 and (**c**) Group 3.

The geometry of the fatigue specimen is shown in Figure 2; it is a kind of standard axial stress fatigue specimen (GB/T 15248-2008) according to the fixture size of the fatigue testing machine and the specimens were prepared by turning process. The diameter of the middle arc segment was 6 mm and surface roughness was controlled by machining parameters. The fatigue specimens were prepared by turning with a cemented carbide blade VBET160408-NGF. The rake angle and the back angle of the blade used were 0 degree and 5 degrees. The radius of the tool tip arc and the edge were 0.8 mm and 40 µm. Emulsion cooling was used for colling during cutting process. *n* is the spindle speed, *f* is the feed rate and *a*_p_ is the cutting depth. Figure 3 shows the CNC (computerized numerical control) turning machine (T-7, LEADWELL Co. Ltd., Taichung, China) which was employed to prepare the fatigue specimens. Table 2 shows the detail processing parameters which were used to process the specimens with the target surface roughness values of *R*_a_ 0.4 μm, *R*_a_ 3.2 μm and *R*_a_ 6.4 μm, respectively.

### 2.3. Surface Integrity Characterization

Surface roughness was measured by a standard profile measuring instrument (Marsurf XT20, Mahr Co. Ltd., Esslingen, Germany). During the surface roughness measurement, the sampling length was 0.8 mm and the evaluation length was 5.6 mm. The measurements were carried out 3 times along the circumference for each workpiece to reduce the measurement error. The average value was taken as the final roughness. In addition, MATLAB (R2020a, MathWorks Co. Ltd., Natick, MA, USA) was used to program and calculate the surface morphology stress concentration factor (*K*_st_) of each sample, which was used to evaluate the degree of micro stress concentration caused by surface micro profile valleys. The calculation method proposed by Arola [22] was used, as shown in Equation (1). The residual stress distribution was measured by X-ray stress test and analysis system (LXRD MG2000, Proto Co. Ltd., Ottawa, ON, Canada). V Kα radiation was used on the {2 1 1} planes under 25 mA and 30 kV. A 1 mm diameter spot was used in the test. During the test, the exposure time and the number of exposures were set to 1 s and 10 times. To measure the stress depth distribution, the specimens were electrolytic-polished for removal of the material layer by layer. After each polish, the polished depth was measured and the residual stress was tested at this depth in the polished area. The polish process was repeated until the residual stress on the new surface was reduced to about 0 MPa. The polishing fluid was saturated with salt water. Microhardness was tested by a microhardness measuring instrument (FEM-8000, FUTURE-TECH Co. Ltd., Hangzhou, China). The measurement standard was Vickers hardness (HV), which uses the length of indentation to evaluate the hardness of material. The measurement parameters of the hardness tests were the weight load of 25 g and the holding time of 10 s, in this work. In addition, to measure the hardness depth distribution, the cross-section sample was cut from the specimen and polished to a smooth surface. The horizontal and vertical distance between adjacent test points were 20 μm and 10 μm. The tests were performed three times at each depth.

(1) $$K_{\mathrm{st}}=1+n\left(\frac{R_{a}}{\bar{\rho }}\right)\left(\frac{R_{y}}{R_{z}}\right)$$

where *n* represents the stress state of the specimen surface; when the specimen surface bears shear load, *n* = 1 and, when the specimen surface bears tensile or bending load, *n* = 2. *R*_a_, *R*_y_ and *R*_z_ belong to the height parameters in the surface roughness characterization system. $\bar{\rho }$, obtained by averaging the three smallest values of one set of profile valley radius *ρ*, is the equivalent profile valley radius. MATLAB was used to fit the profile valley radius *ρ* by using the least square method.

### 2.4. Fatigue Test Setup

The specimens machined by different process parameters were tested on a high temperature universal testing machine (MTS-100kN-13, MTS Co. Ltd., Eden Prairie, MN, USA). The fatigue tests were carried out in accordance with the axial fatigue test standard of China (GB/T 15248-2008). The stress was axially loaded on the fatigue specimen with triangular stress wave. In this work, the maximum stress was 300 MPa, the stress ratio was −1 and the frequency was 1 Hz. The specimens were heated and kept at 550 °C during the fatigue experiment. The upper limit of the fatigue cycle was set to 10^5^ times for all the specimens.

## 3. Results and Discussion

### 3.1. Surface Topography

In order to further clarify the difference in the surface morphology of the three groups of samples, the surface topography was measured by the profile measuring instrument. Figure 3 shows the surface texture measurement results of groups 1, 2 and 3. The texture of wave crests and troughs caused by the increase of turning parameters can be clearly observed. The feed rate *f* was approximately equal to the distance between the two wave crests. After the turning process with the processing parameter of level 1, the surface topography was relatively smooth, as shown in Figure 3a. The surface texture had no obvious fluctuations, which is generally considered as the optimal surface morphology. However, the surface topography of group 2 and 3 showed significant periodic fluctuation characteristics, as shown in Figure 3b,c. The regular wave crests and wave troughs were alternately distributed in the feed direction after the turning process of groups 2 and 3. The processing damages were more likely to appear at these wave crests and troughs, which are considered harmful to fatigue life. The surface texture has a significant relationship with the feed rate *f* and depth of cut *a*_p_. When the feed rate and depth of cut were small (processing parameter level 1), the material removal rate was low, so the residual volume on the surface was dense and short. On the contrary, when the feed rate and depth of cut were big (processing parameter level 3), the residual volume on the surface was rough and high due to the material removal rate being high.

In this work, a total of nine specimens were divided into three groups for processing. Each group of three specimens corresponds to a set of process parameters in Table 2. No. 1–No. 3 samples belong to group 1, No. 4–No. 6 samples belong to group 2 and No. 7–No. 9 samples belong to group 3. Table 3 shows the measurement results of each specimen. It can be seen that the Ra values of the three groups of specimens are basically near the target roughness.

**Figure 4:**
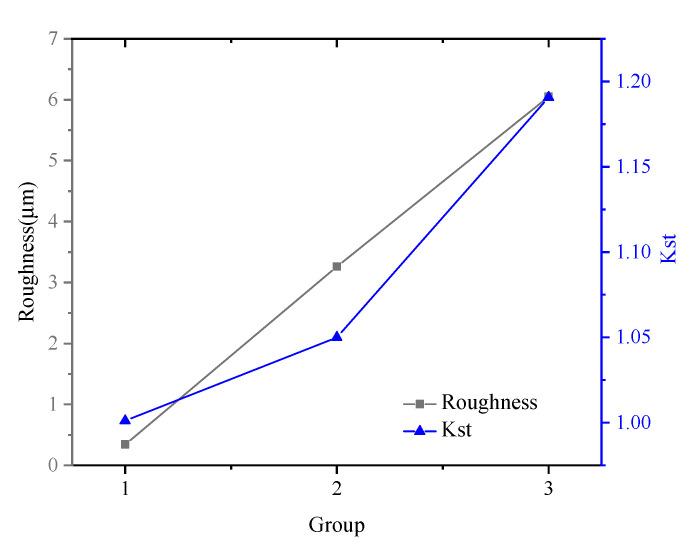
Variation of roughness *R*_a_ and *K*_st_ with turning parameters.

Figure 4 shows the relation between turning parameters and *R*_a_, *K*_st_. It can be seen that the stress concentration coefficient of surface roughness increases with the increase in roughness height parameter *R*_a_. In general, there is no absolute correlation between surface roughness and *K*_st_. The larger surface roughness is, the smaller the curvature radius of the contour valley *ρ* is and the larger the *K*_st_ value is. The value of *K*_st_ is determined by the roughness height parameter and the curvature radius of the valley bottom in the meantime. In this experiment, due to the large difference of roughness values among the three groups of samples, the influence of curvature radius at the bottom of the profile valley was weakened. Therefore, the influence of surface geometric characteristics on fatigue performance was not obvious in this experiment.

### 3.2. Residual Stress Distribution

**Figure 5:**
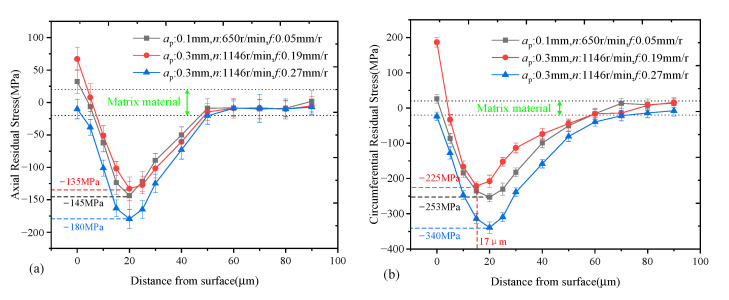
Residual stress distribution of the specimens machined by different processing parameters: (**a**) axial residual stress distribution and (**b**) circumferential residual stress distribution.

Figure 5 shows the measurement results of residual stress distribution of the specimens processed by different parameter levels. The comparison of axial and circumferential residual stress distribution are shown in Figure 5a,b. In the turning process, the generation of residual stress depends on the coupling effect of cutting heat and cutting force. In this paper, a cosine decay function was used to fit the gradient distribution of machining residual stress influence layer:

(2) $$\sigma \left(h\right)=A\cdot e^{\lambda h}\cos\left(\omega h+\theta \right)+\sigma_{0}$$

where *σ* represents the residual stress value, *A* is the amplitude of the cosine decay function, *e* is a constant with the value of 2.718, *λ* is the decay coefficient, ω is the angular frequency and *θ* is the initial phase; *h* is the depth value of residual stress and *σ*_0_ is the matrix material residual stress. Based on the distribution of surface residual stress, Equation (2) was used for fitting to obtain those parameters (Table 4). The fitting process converged quickly and the fitting accuracy was good.

It can be seen that the residual stress was generally distributed in a spoon shape. As the distance from surface increased, both axial and circumferential residual stresses decreased rapidly from the tensile stress (or low compressive stress) to the maximum compressive stress values. After reaching the maximum compressive stress, the residual stress gradually returned to the stress-free state. According to the measurement results, the influence depth of the turning process was about 50 μm for the axial residual stress and it was about 60 μm for the circumferential residual stress. For all specimens, the depth corresponding to the maximum compressive stress was around 20 μm. However, there existed a clear difference in the values of the maximum compressive stress values for the specimens machined by different parameters. The maximum compressive stress of group 3 was significantly greater than that of group 1 and group 2; it reached −179 MPa in the axial direction and −339 MPa in the circumferential direction. Especially in the circumferential direction, the maximum compressive stress of group 3 exceeded the load stress of the fatigue test, which had a significant impact on the growth behavior of fatigue cracks. In addition, another difference should be noted in this study. The surface of the group 3 specimens showed a low compressive residual stress state, while that of the other two groups showed a tensile residual stress state. The influence of this difference is discussed in the next Section. The rate of decay to matrix residual stress is arranged from large to small as the group 1, the group 2 and group 3, respectively. Compared with the value of parameter *λ* in the residual stress fitting aquation of the three groups of specimens, the absolute value of the group 1 was the largest and its attenuation speed was the fastest.

### 3.3. Micro Hardness Distribution

**Figure 6:**
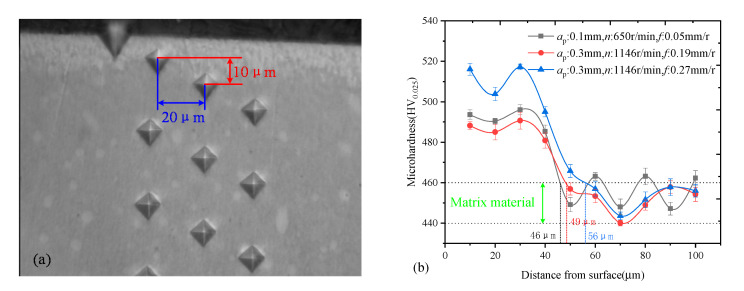
Micro hardness test results of the specimens with different processing parameters (**a**) microhardness distribution test (**b**) microhardness gradient distribution.

Figure 6 shows the measurement results of micro hardness distribution. A series of hardness values were measured with the interval of 10 μm. It can be seen that the three types of specimens have basically the same hardness distribution law. The hardness value was the highest near the surface. As the depth increased, the hardness value gradually decreased until it reached the same hardness as the material matrix. In the matrix area, the hardness measurement results were around 450 HV. In the near surface area, the hardness measurement results were around 500 HV, which was only about 11% higher than the matrix hardness value. The depth of the work-hardened layer was about 40 μm, according to the measurement results in this study. The processing intensity was significantly increased from the processing parameter level 1 to the processing parameter level 3. The depth of work hardening layer increased slightly in turn. This result suggests that the surface hardness of the Ti_2_AlNb alloy can only be slightly improved during the turning process. The turning process parameters have no significant influence on the micro hardness distribution of the Ti_2_AlNb alloy specimen.

### 3.4. Fatigue Life and Fracture

**Figure 7:**
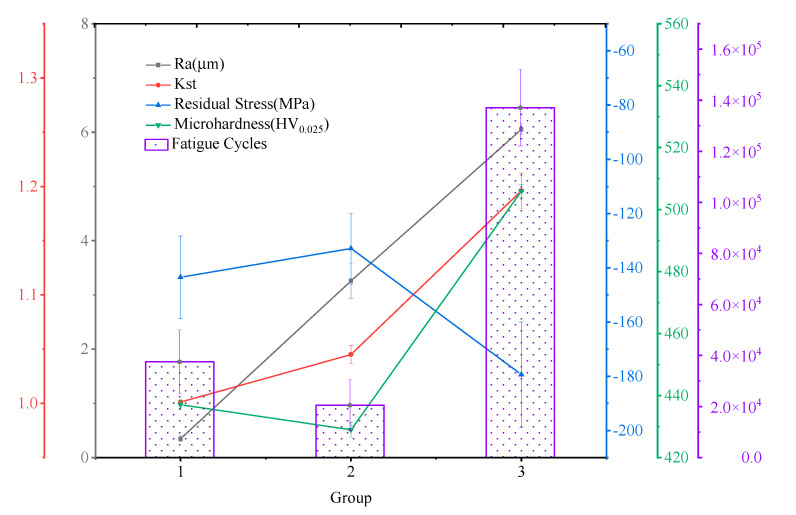
Fatigue test results of the specimens with different surface roughness.

Figure 7 shows the hot fatigue test results of the specimens machined by different parameter sets. It can be seen that there was an interesting trend in fatigue life. Fatigue life decreased first, then increased during the increasing process of surface roughness. The specimens with the largest surface roughness obtained the largest fatigue cycles. Generally, there existed machining damage and crack sources in surface layer with large surface roughness value. Therefore, it was concluded, in some studies, that the greater the surface roughness, the shorter the fatigue life within a certain range of *R*_a_ values [13]. However, the hot fatigue test results suggest that there were some factors other than surface roughness that played a key role in the fatigue behavior of the Ti_2_AlNb alloy [17,18]. The fatigue cycles of the group 3 specimens were significantly greater than those of the group 1 and group 2. This result shows that other factors have a more significant influence on fatigue life than surface roughness. According to the results in Section 3.2 and Section 3.3, it can be concluded that the residual stress and microhardness had significant effect on the fatigue behavior of the specimens. The residual compressive stress offset part of the tensile load and the existence of a hardened layer increased the stress threshold of crack generation and development, then significantly improved fatigue life under thermomechanical load.

**Figure 8:**
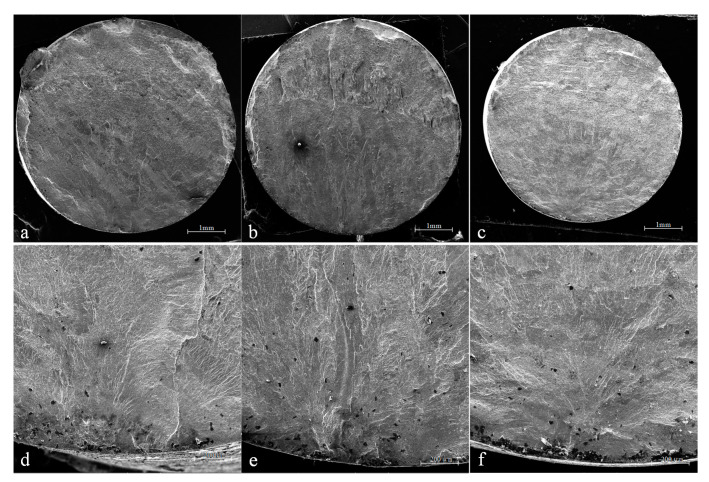
Fatigue fractures of the different specimens. (**a**) Overall fracture morphology and (**d**) crack source characteristics of Group 1 specimens, (**b**) overall fracture morphology and (**e**) crack source characteristics of Group 2 specimens, (**c**) Overall fracture morphology and (**f**) crack source characteristics of Group 3 specimens.

Figure 8 shows the fatigue fractures of the different specimens. The fractures were observed by SEM. It can be seen that all fractures present the characteristics of single-source fatigue fracture. The fatigue cracks started from the surface and expanded radially to the substrate of the specimens. Even for the specimen No. 8, which had a large roughness value, the fatigue fracture was a single-source feature. This result indicates that surface roughness had no significant influence on the hot fatigue behavior of the Ti2AlNb alloy.

**Figure 9:**
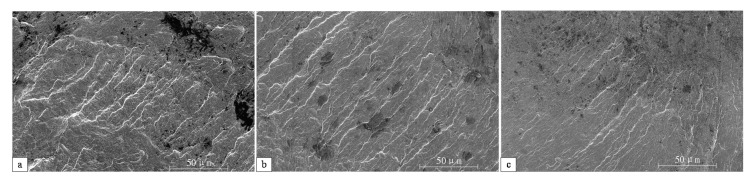
Fatigue strips of the fractures. (**a**) Group 1; (**b**) Group 2; (**c**) Group 3.

In order to find out the reason for the difference in fatigue life, the local area of the fatigue fractures was further observed by SEM. Figure 9 shows the characteristics of the fatigue fractures which were observed near the crack source. The fatigue fractures reflected the macroscopic characteristics of fatigue crack growth. It can be seen that the fatigue fractures of different specimens were significantly different. The fatigue fractures of specimens No. 2 and No. 5 had obvious texture characteristics, but the fatigue fractures of specimen No. 8 had only slight texture characteristics. According to Figure 9, the spacing between the fatigue fractures was approximately 4.6 μm, 5.3 μm and 2.7 μm for specimens No. 2, No. 5 and No. 8, respectively. This result indicates that the growth of fatigue cracks was suppressed in specimen No. 8. According to the measurement results of the residual stress distribution in Figure 5, the high residual compressive stress in the surface layer was considered to be the main factor in inhibiting crack propagation. In specimen No. 8, the compressive stress was distributed from the surface to 60 μm below the surface. Especially, the maximum compressive stress reached −179 MPa in the axial direction and −339 MPa in the circumferential direction. In this work, the load stress of the fatigue test was 300 MPa (the detail has been provided in Section 2.4). In specimen No. 8, the residual compressive stress significantly offset the tensile stress load during the fatigue test. The result in Figure 9 indicates that the strong compressive stress distribution had a great inhibitory effect on crack propagation in the Ti_2_AlNb alloy.

Generally, the greater the surface roughness, the more the surface damages and the shorter the fatigue life. However, the hot fatigue test results in this work show that specimens No. 7, No. 8 and No. 9 obtained the largest fatigue cycles. The surface of these specimens was quite rough, but there existed a strong residual compressive stress and good hardened layer in the surface layer of the specimens. Therefore, the compressive residual stress and machined hardened layer play important roles in the hot fatigue behavior of the Ti_2_AlNb alloy.

## 4. Conclusions

(1) The greater the processing parameters, the greater the Ra values and the *K*_st_ values of the specimens. The hot fatigue test results in this work show that the fatigue life of the Ti2AlNb alloy was not sensitive to surface roughness.

(2) The residual stress distribution showed a spoon shape in the Ti_2_AlNb specimens after the turning process. As the distance from the surface increased, the residual stress decreased rapidly to the maximum compressive stress. After reaching the maximum compressive stress, the residual stress gradually returned to the stress-free state. The surface hardness of the Ti_2_AlNb alloy could only be slightly improved during turning process.

(3) The residual stress and microhardness had a significant effect on the fatigue life of the Ti_2_AlNb alloy. Even when the surface of the specimen was very rough, the largest fatigue cycle could still be obtained. The present results suggest that more attention should be paid to the residual stress and microhardness than surface roughness in the application of the Ti_2_AlNb alloy.

## Figures and Tables

**Table 1:** Major contents of Ti_2_AlNb.

Element	Al	Nb	Mo	Zr	Ti
wt.%	10.95	37.74	1.79	1.67	Other

**Table 2:** The processing parameter sets correspond to different target roughness.

Parameters	Level 1	Level 2	Level 3
*a*_p_ (mm)	0.1	0.3	0.3
*n* (r/min)	650	1146	1146
*f* (mm/r)	0.05	0.19	0.27

**Table 3:** The processing parameter sets correspond to different target roughness.

Specimen No.	*R*_a_ (μm)	*R*_y_ (μm)	*R*_z_ (μm)	*ρ* (μm)	*K*_st_	Group
1	0.3964	2.0512	2.0194	697.6331	1.0012	Group 1 *a*_p_, 0.1 mm; *n*, 650 r/min; *f*, 0.05 mm/r
2	0.3427	2.029	1.9891	717.5242	1.0010	
3	0.3752	2.0905	2.0496	706.1372	1.0011	
4	2.9380	13.257	13.1901	143.5176	1.0412	Group 2 *a*_p_, 0.3 mm; *n*, 1146 r/min; *f*, 0.19 mm/r
5	3.2589	13.0042	12.9865	130.9969	1.0498	
6	3.0105	13.601	13.5711	136.4176	1.0442	
7	6.1271	26.5943	26.7299	59.4716	1.2050	Group 3 *a*_p_, 0.3 mm; *n*, 1146 r/min; *f*, 0.27 mm/r
8	6.0504	25.1128	24.9831	64.4499	1.1887	
9	6.0255	25.8038	25.9345	61.6457	1.1944	

**Table 4:** Key parameter values of residual stress fitting model.

No. Specimen	*A*	*λ*	*ω*	*θ*
2	6.889	−4.615	1.590	1.555
5	26.893	−3.929	0.357	1.569
8	2.175	−3.368	4.783	1.510

## Data Availability

The data presented in this study are available on request from the corresponding author. The data are not publicly available due to funder data retention policies.
